# Factors influencing timely initiation and completion of gestational diabetes mellitus screening and diagnosis - a qualitative study from Tamil Nadu, India

**DOI:** 10.1186/s12884-017-1429-y

**Published:** 2017-08-01

**Authors:** Karoline Kragelund Nielsen, Thilde Rheinländer, Anil Kapur, Peter Damm, Veerasamy Seshiah, Ib C. Bygbjerg

**Affiliations:** 1 Department of Public Health, University of Copenhagen, Oester Farimagsgade 5, 1014 Copenhagen, Denmark; 2 Steno Diabetes Center Copenhagen, Niels Steensens Vej 6, 2820 Gentofte, Denmark; 3 World Diabetes Foundation, Brogaardsvej 70, 2820 Gentofte, Denmark; 4 Center for Pregnant Women with Diabetes, Department of Obstetrics, Rigshospitalet, The Institute of Clinical Medicine, Faculty of Health and Medical Sciences, University of Copenhagen, Blegdamsvej 9, 2100 Copenhagen, Denmark; 5 Dr. Seshiah Diabetes Research Institute and Dr. Balaji Diabetes Care Centre, 729 Poonamallee High Road, Aminjikarai, Chennai, Tamil Nadu 600029 India

**Keywords:** Gestational diabetes, Screening, India, Health care services, Antenatal care, Guidelines, Qualitative research

## Abstract

**Background:**

In 2007, universal screening for gestational diabetes mellitus (GDM) was introduced in Tamil Nadu, India. To identify factors hindering or facilitating timely initiation and completion of the GDM screening and diagnosis process, our study investigated how pregnant women in rural and urban Tamil Nadu access and navigate different GDM related health services.

**Methods:**

The study was carried out in two settings: an urban private diabetes centre and a rural government primary health centre. Observations of the process of screening and diagnosis at the health centres as well as semi-structured interviews with 30 pregnant women and nine health care providers were conducted. Data was analysed using qualitative content analysis.

**Results:**

There were significant differences in the process of GDM screening and diagnosis in the urban and rural settings. Several factors hindering or facilitating timely initiation and completion of the process were identified. Timely attendance required awareness, motivation and opportunity to attend. Women had to attend the health centre at the right time and sometimes at the right gestational age to initiate the test, wait to complete the test and obtain the test report in time to initiate further action. All these steps and requirements were influenced by factors within and outside the health system such as getting right information from health care providers, clinic timings, characteristics of the test, availability of transport, social network and support, and social norms and cultural practices.

**Conclusions:**

Minimising and aligning complex stepwise processes of prenatal care and GDM screening delivery and attention to the factors influencing it are important for further improving and expanding GDM screening and related services, not only in Tamil Nadu but in other similar low and middle income settings. This study stresses the importance of guidelines and diagnostic criteria which are simple and feasible on the ground.

**Electronic supplementary material:**

The online version of this article (doi:10.1186/s12884-017-1429-y) contains supplementary material, which is available to authorized users.

## Background

Gestational diabetes mellitus (GDM) increases the risk of various adverse pregnancy outcomes, as well as the risk of future diabetes in both the mother and offspring [1–4]. According to estimates from the International Diabetes Federation, approximately 21.4 million live births are annually affected by hyperglycaemia in pregnancy of which 84% are affected by GDM [5]. Despite the increased focus on GDM screening and diagnosis in recent years, also in low and middle income countries [6–8], very little is known about the barriers and challenges faced by women in this process, and how to implement or improve GDM screening and care on a large scale in such settings [9].

Based on work on diabetes and chronic disease management, *Mol* [10] showed that what may seem a simple process – the patient being informed about the use of a medical technology (e.g. blood sugar measurement), which he/she then uses to monitor his/her disease – is far from straightforward. *Mol* argues that the problem is not in the decision-making process (the patient was given the information and choice to do the blood sugar measurement or not), but in actually ‘doing’ the blood sugar measurement [10]. In order for the ‘doing’ to take place, the circumstances have to be right, and the technology has to be appropriate for the patient [11]. These issues are rarely taken into consideration when evaluating the use of diagnostic devices or test procedures and receive little attention despite the significant impact they have [11]. Thus, applying *Mol’s* notions of ‘doing’ to analyse the GDM screening process can provide us with a deeper understanding of women’s GDM screening experiences and behaviours.

India and in particular the South Indian state of Tamil Nadu (TN) is a highly relevant setting for such analysis: 65 million people in India are estimated to have diabetes [5] and the country continues to struggle with high maternal mortality and morbidity, accounting for 19% of worldwide maternal deaths [12]. A high prevalence rate of GDM (14%) has been identified in TN, and has subsequently been noted in other areas of India as well [13–15]. This led the Government of TN to introduce GDM screening as part of routine antenatal care (ANC) services at government health centres in 2007, applying the criteria recommended by the Diabetes in Pregnancy Study Group India (DIPSI) [16], i.e. a single-step test using a 75 g oral glucose tolerance test (OGTT) with diagnosis being made if the two hour value is 140 mg/dl or more. Screening for GDM is typically done in the 24–28th weeks of gestation, but sometimes also at the first ANC visit and it may also be repeated later in pregnancy. Additionally, awareness campaigns and capacity building initiatives targeted at both the government and the private health care system were carried out.

The health care system in TN as in rest of India is complex; involving a mixture of public and private health care providers (HCPs), practising different systems of medicine (modern, Ayurveda, Unani and Siddha). In TN, only 53% of the population uses the public health sector; with higher rates in rural settings, and among the poor [17]. The organisation of ANC services is also complex, with different levels of services, facilities and specialisations in rural and urban areas, and in the public and private sector. There are no clear-cut referral systems, and women’s use of ANC services depend on their practical access, willingness and ability to pay for services. Many women attend both public and private health care services [18]. Other factors that determine utility of maternal health services in India include women finding the service relevant to themselves and their fetus/child, quality of care [19], their socio-economic status [20–22], distance to health facility, literacy, parity, obstetric history [23], husband’s knowledge and attitude towards services [24], family traditions, financial constraints, behaviour of HCPs [25], and women’s autonomy [26]. Thus, accessing and utilising services is much more complex than merely a question of awareness.

Implementing timely and effective detection and management of GDM in TN requires a good understanding of how women navigate their way through the complex health system. Therefore, our study investigated factors that might influence timely initiation and completion of the GDM screening and diagnosis process in a sample of urban and rural women in TN.

## Methods

### Study area

The study was carried out in two areas of TN: Chennai city and Thiruvallur district representing two different settings for GDM screening: a private urban and a government rural centre [27].

Chennai is the state capital, is currently experiencing substantial economic growth and is home to a growing middle class. In Chennai city, the study was conducted at a private diabetes hospital catering mainly to the urban middle class.

In Thiruvallur district, the study was conducted at a government primary health centre and its surroundings, located in a semi-rural area approximately 40 km north of Chennai city. However, most pregnant women attending ANC here reside in rural areas. While some inhabitants in the area work in Chennai, many are farmers, daily wagers or workers at the local factories. The villages are connected to the main roads via small paved or dirt roads.

### Data collection, analysis and ethical considerations

Data for this study was collected between January 2013 and September 2014 as part of a larger study on GDM [27].

The study was qualitative with triangulation of several data sources and methods: observations at health facilities, and interviews with pregnant women and HCPs. This approach aimed at providing a holistic picture of the investigated phenomenon, while increasing validity and reducing the risk of bias [28].

Around 120 h of observation were conducted (by the lead author KKN) at the two health centres, with a focus on the screening process and patterns of actions as well as verbal and non-verbal interactions. Detailed note-taking was done. Observations provided contextual knowledge about the health system and organisation of services and highlighted issues from a third party perspective that informants (users and provider of services) take for granted and therefore do not mention in interviews [29].

Semi-structured interviews were conducted with 30 pregnant women attending ANC at the rural government primary health centre (18 women) and the private urban diabetes centre (12 women). An overview of the women is presented in Table 1. A purposive sampling strategy was used, and all the pregnant women had or were supposed to have undergone GDM screening procedures at the health centres during their current pregnancy. They were recruited at the health facility, through the local village health nurses or through referral from other pregnant women. Recruitment was continued until theoretical saturation was reached. The interviews took place at a private area of the health centre or at the women’s homes and focused on their experiences with and perceptions of GDM screening services (Additional file 1).

**Table 1:** Characteristics of the pregnant women interviewed

	Area	Age	Education	Gestational age at first test
Woman 1	Urban	35	Graduate	2.5 months
Woman 2	Urban	23	12th Grade	4.5 months
Woman 3	Urban	31	Postgraduate	1.5 months
Woman 4	Urban	29	Postgraduate	1.5 months
Woman 5	Urban	23	Graduate	2 months
Woman 6	Urban	26	Postgraduate	2 months
Woman 7	Urban	25	Postgraduate	1 month
Woman 8	Urban	20	12th Grade	9 months
Woman 9	Urban	38	8th Grade	2 months
Woman 10	Urban	28	Postgraduate	3 months
Woman 11	Urban	25	Postgraduate	1 month
Woman 12	Urban	26	Graduate	3 months
Woman 13	Rural	28	12th Grade	8 months
Woman 14	Rural	25	8th Grade	7 months, but did not complete
Woman 15	Rural	23	9th Grade	5 months
Woman 16	Rural	22	10th Grade	7 months
Woman 17	Rural	21	10th Grade	3.5 months
Woman 18	Rural	22	Graduate	3 months, but did not complete 6 months when it was completed
Woman 19	Rural	22	9th Grade	4 months
Woman 20	Rural	20	7th Grade	4 months
Woman 21	Rural	22	10th Grade	5 months, but did not complete
Woman 22	Rural	24	Postgraduate	6 months
Woman 23	Rural	23	12th Grade	7 months
Woman 24	Rural	25	10th Grade	4 months, but did not complete 6 months when it was completed
Woman 25	Rural	26	12th Grade	7 months
Woman 26	Rural	25	10th Grade	4 months, but did not complete 5 months when it was completed
Woman 27	Rural	25	8th Grade	6 months
Woman 28	Rural	23	10th Grade	5 months
Woman 29	Rural	22	10th Grade	4 months
Woman 30	Rural	21	10th Grade	3 months

Additionally, interviews with nine HCPs were carried out (four laboratory technicians, two village health nurses, one physician, and two private practicing gynaecologists/obstetricians). These HCPs were selected based on having a crucial role in the screening process. The interviews focused on their experiences in providing GDM screening services and challenges and opportunities in doing so (Additional file 2). A few village health nurses approached for interview declined, stating lack of time as the reason.

Informants fluent in English were interviewed by KKN, but the majority of interviews were conducted with support of a trained research assistant fluent in both English and Tamil. The overall purpose of the study was described to the participants, and verbal informed consent obtained prior to the interview, including approval for audio recording the interview. Verbal consent was chosen over written consent as obtaining written consent was seen as counterproductive to the informal and comfortable atmosphere we were trying to create to facilitate rapport and building trust between the researchers and informants [30]. The verbal informed consent was given in the presence of KKN and the research assistant. Ethical approval for the study, including the use of verbal informed consent, was given by the Institutional Ethics Committee at Dr. Seshiah Diabetes Research Institute and Dr. Balaji Diabetes Care Centre in Chennai.

**Fig. 1:**
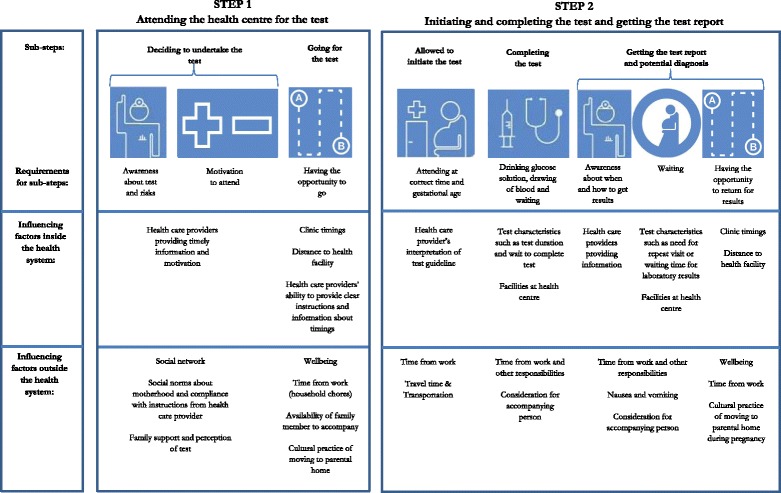
Steps, sub-steps, and requirements in the GDM screening and diagnosis process, and influencing factors within and outside the health system

The interviews, most lasting between 30 and 60 min, were transcribed verbatim and translated into English. The interview guides for the research intended to explore a number of overall topics, e.g. experience with the process, social support and informants’ knowledge and expectations of the GDM test. An iterative-inductive analysis process [31] was followed, starting during fieldwork, which allowed follow-up and collection of additional data on emerging issues. Qualitative content analysis of interview and observation data was done by KKN, who read through and organised data into meaning units, categories and themes [32]. The coding was done using the software programme NVivo 10 (QSR International Pty Ltd. 1999–2012). First step of the analysis was to describe the differences in GDM screening and diagnosis procedures and compare these with government order instructions. Next step of the analysis identified two overall steps with two and three sub-steps, respectively. At each sub-step there were certain requirements or ‘doing’ as well as factors influencing women’s navigation of the process (Fig. 1).

## Results

### Procedures for GDM screening and diagnosis

Data from observations and interviews with HCPs was synthesised on the actual procedures of GDM screening in the urban and rural health systems and compared to government order instructions on screening for hyperglycaemia in pregnancy (see Table 2). This analysis revealed several differences in GDM screening approaches: 1) different testing ‘procedures’ 2) different timing of GDM testing and 3) different diagnostic criteria and procedures.

**Table 2:** Procedures for GDM screening and diagnosis synthesised based on observations, interviews, and available written information, versus instructions from government order on screening

		Private urban centre	Government rural centre - performed by private-non-profit laboratory technician	Government rural centre -performed by government laboratory technician	Government order on screening for hyperglycaemia in pregnancy
Step 1: Attending the health centre for the test	*Informed and referred for the test*	Via the gynaecologist/obstetrician	Via the village health nurse	Via the village health nurse	Not mentioned
Step 2: Initiating, completing and getting the report of the test	*Attending days*	Fasting on Mondays-Saturdays	Fasting on Tuesdays	No-fasting on Thursdays	1) Irrespective of whether she has taken her breakfast or not
	*Attending time*	Morning	Morning	Morning	2) Not mentioned
	*Gestational age at attendance*	No specific gestational age required, but testing is recommended in each trimester	No specific gestational age required, but testing is recommended in each trimester	4th, 6th and 8th month of pregnancy	3) Gestational week 24–28
	*Flow of the testing procedure*	1) Register 2) Consultation with doctor 3) Pay bill 4) Give fasting venous sample 5) Drink prepared glucose solution 6) Wait 1 h 7) Give 1 h venous sample 8) Wait 1 h 9) Give 2 h venous sample 10) Have breakfast and wait for results	1) Register 2) Give fasting venous sample 3) Mix and drink glucose solution 4) Wait 1 h 5) Give 1 h capillary sample 6) Wait 1 h 7) Give 2 h venous sample 8) Go home or wait for regular ANC check-ups	1) Register 2) Drink prepared glucose solution 3) Wait 2 h 4) Give 2 h venous sample 5) Go home	4) Registration not mentioned 5) Drink 75 g glucose solution 6) Wait 2 h 7) Give 2 h venous blood sample
	*Getting the test report*	On the day of the test. Around 1.5–2 h after 2 h sample	Usually 1 week later	If plasma glucose value is 140 mg/dl or more laboratory technician informs village health nurse who informs woman to come back fasting for a second test	Not mentioned
Diagnosis	*Steps of confirming test/s step to be completed*	A second test not requested as diagnosis is based on first test	A second test not requested as diagnosis is based on first test	1) Register 2) Fasting venous blood sample 3) Have breakfast consisting of 4 idlys (lentil and rice cakes). 4) Wait 1.5 h 5) Give 1.5 h venous sample 6) Go home	A second test not requested as diagnosis is based on first test
	*Diagnostic cut-off levels used*	2 h plasma glucose value ≥140 mg/dl (7.8 mmol/l)	2 h plasma glucose value ≥140 mg/dl (7.8 mmol/l)	Fasting plasma glucose value ≥110 mg/dl (6.1 mmol/l) 1.5 h plasma glucose value ≥140 mg/dl (7.8 mmol/l)) Both values have to be above the cut-off	2 h plasma glucose value ≥140 mg/dl (7.8 mmol/l)

At the rural government centre, GDM screening was carried out by a government employed laboratory technician as well as by a private-non-profit employed laboratory technician, working for a larger study on GDM (see Balaji et al. 2014). As shown in Table 1, the GDM screening process differed somewhat between the two categories of laboratory technicians at the rural centre. For testing by the government laboratory technician, the women had to attend in the fourth, sixth and eight months of pregnancy, whereas no specific gestational age was required for testing by the private-non-profit rural laboratory technician or at the urban site. A 75 g OGTT was used at all sites. A fasting venous sample was collected both at the urban centre and by the private-non-profit employed laboratory technician at the rural centre. At these two sites plasma glucose was also estimated at one and two hours after intake of the glucose solution. At the urban centre both of these were measured in venous samples, whereas the private-non-profit employed laboratory technician at the rural centre measured the one hour plasma glucose with a calibrated glucometer (capillary sample), and the two hour measurement was on venous blood sample. The government employed laboratory technician only took one sample: a two hour venous sample after ingestion of 75 g glucose load. At the urban centre the women were asked to wait for the results of their test and were given the results the same day, whereas women attending the rural centre got the results after a couple of days to weeks later. These differences are important and have consequences for large scale implementation as described later.

Analysis of the observational and interview data showed that at all sites two main steps had to be completed in order to ensure timely screening and diagnosis: 1) attending the health centre, and 2) initiating, completing and getting the test report. The government employed laboratory technician at the rural centre required an extra step where a second test was administered; which meant a repetition of the two main steps (Table 2). Each step had sub-steps and certain requirements or ‘doing’ for completion, and various factors outside and within the health system influenced whether such ‘doing’ took place (see Fig. 1 and Table 3).

**Table 3:** Barriers and facilitators for completing the two steps of the GDM screening process

Steps	Barriers	Facilitators
Step 1 Attending the health centre	• Not being informed (timely) about test by HCP • Weak or lack of social network	• Being informed (timely) about test by HCP • Strong social network • Strong motivation • Awareness of risk • Social norms about pregnancy and motherhood • Belief in importance of compliance with instructions from health care providers
	• Unsupportive family • Work • Feeling unwell • Being away from home	• Family in support of test • Conducive clinic timings
	• Lack of family member to accompany or help with household work	• Family member to accompany or help with household work
Step 2 Initiating, completing and getting the report of the screening and diagnostic test in a timely manner	• Not being aware of test time schedule • Lack of or delayed transportation and heavy traffic • No help with household work • Inflexible interpretation of timing of test in terms of gestational age	• Awareness of test time schedule • Available and timely transportation and conducive traffic • Help with household work
	• Glucose solution eliciting vomiting • Procedure stating that test has to be cancelled and done another day when women vomit	• Adding lemon drops to glucose solution
	• Long waiting time • Uncomfortable physical ambience of health centre and attitude of staff • Health care provider not informing the women about how and when the results will be available • Health care provider not informing the women about the results within a reasonable time period • Barriers mentioned under step 1 when women have to return to the health centre again	• Comfortable physical ambience of health centre and attitude of staff • Health services being free of charge • HCP informing the women about how and when the results will be available • HCP providing the results to the women over the phone or in-person in the village

Overall, interview data showed that women’s experiences of the screening process often differed based on rural or urban contexts. This was partly influenced by the differences in the screening procedures, but also by differences in infrastructure and access, and socio-economic backgrounds of the women as described in the following sections.

### Step 1: Attending the health centre for the test

The first step in the process had two sub-steps: 1.a deciding to undertake the test and 1.b actually going for the test.

#### 1.a). Deciding to undertake the test: Awareness and motivation

The analysis of the interviews with pregnant women and the HCPs identified that being *aware* of the test, including whether and why one had to undertake it, was the first requirement for deciding to go for the test. In practice, this meant that the pregnant women had to receive (correct) information about the test. This was usually done by the HCPs, i.e. the gynaecologist/obstetrician, if the women attended the urban diabetes centre, and the local village health nurse or the laboratory technician, if the women attended the rural government primary health centre. According to HCPs, this information is always provided, usually at the first ANC visit. However, six of the interviewed women from the rural area said they had not been informed about taking the test from their HCP – or if they had, it was only late in their pregnancy. Most of them had heard about the test from family or neighbours, or from other pregnant women attending ANC at the health centre or at the local *balwadi* (community pre-school and health post). A 26 year old woman from the rural area gave the following account of how she found out about the GDM screening test:

*“Last week when I went for the regular check-up, I saw the other women sitting in a group for some test. So I asked them what test they were taking. They said “sugar test”, and then I said “every month they take it, right? With the regular blood test?” The women said “yes”*. *She [the village health nurse] hasn’t told me anything. She comes here to give us the vaccination and that’s all. We never see her in that hospital. She never helps us… We ourselves go and find out things.”* (Woman no. 25, rural area).

This woman had been tested for GDM for the first time three days before the interview at seven months into her pregnancy. Two other women from the rural area similarly had the first test done in 7th and 8th month, having not been informed about the test earlier. In the rural area, not receiving timely information about the test would thus delay or rule out testing entirely, clearly illustrating how dependent screening is on proper and timely information by HCPs. On the other hand, it also reveals the importance of social network of women, and how they can function as facilitators; in this case for conveying information about the screening test, and, thus, compensate for the shortcomings of the health system. In the urban area, women also relied on their social network such as family and friends – but mainly when choosing obstetrician or maternity clinic, rather than as sources of screening information.

The second requirement for initiating testing was *motivation* to attend for testing. Once aware of the possibility for getting tested, most interviewed women displayed great motivation for the test. The motivation and decision to undertake the test was strongly influenced by the HCPs advice. Around 1/3 of all the women said they did the test mainly because their HCP had sent them for it and because they considered it to be compulsory.

*“The doctor made this report, and that I have followed. Because it was just a blood test. When the doctor was saying “take it” and wrote it in the report and everything, that way I knew [about the GDM screening test]. The doctor has written this, so I follow their rules.”* (Woman no. 3, urban area).

*“I asked her [the village health nurse] what to do. She said that “tomorrow everyone will be doing the test. You too go and do it”.*” (Woman no. 13, rural area).

Motivation was also strongly linked to the fear of the consequences of GDM and a strong desire to do whatever possible for the fetus’ health. By doing the test, women saw an opportunity to prevent a danger at an early stage. Not taking the test was considered hazardous, risking harmful effects to the baby.

*“We should do the sugar test. Only if we do it, we would have a healthy baby… If not, the baby would be born very big… We would have to do a caesarean, and if we have sugar, the place where the stitches are being put, would not heal fast. So we have to do a sugar test to check if we have sugar. Because if we have sugar even the baby would get it. If we do the test it’s for the good of the child.”* (Woman no. 15, rural area).

The prominence of motherhood was also mentioned in many interviews as a motivator for testing. Women stressed their new role as responsible mothers, and how GDM could potentially hamper the delivery of a healthy baby, and thereby risk the discontinuation of the family. Not handling this risk, could also lead to social stigma through gossip, particularly in the rural area, as aptly described by one woman: *“Becoming a mother is a big thing. So then if there is a problem [such as GDM] it would be sad only. It is like going through pain and then having another generation, and then if we don’t deliver the baby well, the neighbours and all would talk badly about us.”* (Woman no. 29, rural area).

Finally, deciding to attend the test was strongly influenced by the family. Almost all women from the rural area and around half of the urban women lived in joint families, typically with their in-laws, as is common in India. Many women in both areas said that their families would encourage and endorse them to undertake not only the GDM screening test, but all other tests and scans required during pregnancy.

*“My parents, my husband, my mother-in-law, father-in-law, they all said “take all the tests, because this is the time to get the whatever, so you should be very careful this time. So whatever the doctor say, you should take that test. Whatever they say, you should follow that”.”* (Woman no. 5, urban area).

Many women explained that they had to seek the approval of their husband and/or mother-in-law before going to the test, who had given it willingly – particularly as the testing was instructed by the HCP as part of routine ANC.

*“For them it was just like “okay this is the protocol now. These tests are done for everybody, so it is good. You can rule it out now or take care from now”. So they have been quite understanding about it as well.”* (Woman no. 4, urban area).

Only two of the interviewed women (women no. 18 and 25, both from the rural area and newly married) gave accounts of how family members had been less enthusiastic about the test. The family was either concerned about the risk of evil spirits when pregnant women travel or about the drawing of blood. Low haemoglobin level is known in Tamil language as having ‘less blood’, and anaemia among women is very prevalent in this population. Thus, ‘drawing of blood’ seemed contradictory to these family members according to the pregnant women. Yet, both women also had family members supporting them in attending the test, and eventually the women disregarded the concerns and attended the GDM screening test. These findings highlight that having a supportive, well-informed family is clearly an important facilitator for GDM testing.

#### 1.b). Going for the test: Having the opportunity to go

Having decided to go for the test, women encountered the first ‘doing’ of the screening procedure: actually *going* for the test. This required they had the opportunity to go, which was influenced by several factors (Fig. 1 and Table 3).

The women’s well-being during pregnancy was one such issue, which could delay going for the test. In interviews, some women from both areas mentioned that pregnant women were not supposed to travel since it could harm the fetus, and a few women explained that they had delayed the test because of vomiting or tiredness. A 28 year old woman, attending the urban centre for her first GDM screening test in the beginning of her third month, said:

*“My gynaecologist told me to go [for the test] by the beginning of the second month. She said to go whenever I feel a little okay and when the nausea is all down and all that. But then it didn’t seem to come down at all, and I was constantly throwing up and I was taking drips. There was no way I could come to this place [health centre] that early in the morning. It was impossible. So I had no chance of coming here for the diabetes test. But now, for the past two weeks, I was a little okay, so we just thought we will finish it off.”* (Woman no. 10, urban area).

Another challenge was coordinating the test with job timings, especially for urban women with formal jobs. One informant, a 26 year old accountant pregnant for the first time, was working nightshifts in a US company in Chennai and said: *“If I have to go for a night shift, I can’t do the test. I won’t have sleep. From morning to the evening I have to sleep, and night I have to go for night shift. So, since I have taken leave, I am feeling relieved.”* (Woman no. 12, urban area).

This woman had taken one week’s unpaid leave in order to rest and finish the required ‘doing’ of pregnancy tests, including the GDM test. The urban centre was also open on Saturdays, and from observations and informal conversations it was clear that this helped many working pregnant women to come for the test (including woman no. 4 and woman no. 6 in this study).

Women without formal jobs had to coordinate testing attendance with household work and child care. Women living in joint households sometimes noted that their family had helped them free up time from household work to go for the test. A couple of women felt bad about asking for such help, and one woman noted that: *“To leave my child at home – I find it difficult.”* (Woman no. 15, rural area). Some rural women therefore brought the child along with them to the health centre, which they were generally allowed to, but which could complicate completion of the second step of screening.

These factors were further aggravated by the fact that many women due to social norms needed to be accompanied by family members when travelling. This meant more coordination and potential delay. The need to have a person accompanying the pregnant woman was described by women as also being practical, e.g. securing transportation, social and for security, e.g. assistance if falling ill.

Difficulties with organising this were reported by a few women in the rural as well as urban settings. One 20-year old pregnant woman reported that it had taken her two months and a lot of arguing with her husband, from when she was advised to attend the test by her gynaecologist, till she could go to the test:

*“I used to shout at him for not taking me. “When will you take me?” To take me here to check the sugar. When he came back from his native place I somehow told him to take me today. So [this] morning he brought me. I used to fight with him. “You take me to the doctor! Take me to the doctor!”.”* (Woman no. 8, urban area).

A final reason not to undertake the test, according to HCPs, was the common Indian practice where women go to live in their parental home sometime during pregnancy. If the parental home was located in another health district, the women would not fall under the care of their usual health centre and could miss the screening notifications from their HCPs: *“Some of them would say that they have gone out of station [left their home], and that they could not do the test… I would tell them to do the test compulsory in the fourth month. Every month when they come for the test, I would check their notebook, and when I ask them why they didn’t come in the fourth month, they would say that they had gone out of station.”* (Government laboratory technician, rural health centre).

However, the interviewed women themselves mainly reported this as a reason why they did not collect the test results.

### Step 2: Initiating, completing and getting the test report

In spite of the identified barriers, all interviewed women had managed to attend the health centre at least once for the GDM screening. Upon arrival at the health centre, the women had to meet other requirements for the sub-steps: 2.a being allowed to initiate the test, 2.b complete the test and 2.c getting the test report (Fig. 1).

#### 2.a). Allowed to initiate the test: Attending at correct time and gestational age

Even if the pregnant women arrived at the health centre, particularly at the rural health centre, there were situations where the women were not allowed to take the test. Observations showed that in the urban centre and in the rural centre, when conducted by the private-non-profit laboratory technician, the test procedure was typically organised to be done early in the morning during a specific timeslot, with the women being fasting. Some women arrived too late and were therefore refused or at least strongly encouraged not do the test, and instead told to come back another day. Interviews provided more knowledge about why some women had problems complying with this aspect of ‘doing’ the test. A 25 year old woman from the rural area, who was first screened when six months pregnant, described how she had attended the test in her fourth month, but was refused the test:

*“I came [for the GDM test], but they told me that “it has become late” and they told me that “we can’t do the test”. So I went back home. I travelled so far, and then when I came here I could not take the test since it was late. Because that day the bus came late.”* (Woman no. 24, rural area).

Thus, transportation issues as well as not being aware of the test timings, and completing household work were other challenges mentioned by women for their inability to reach the centre in time.

One of the HCPs described how he was often faced with women coming too late: *“If they come for the test at 10 o’clock, the test would be over at 12 o’clock, so they have to wait till 12 o’clock. They would say that they will wait, but I can’t wait, because I have to give the samples at the hospital. The other patients would have come at 8 o’clock, so then I need to speak to the patient and make her understand. At that moment the patient would be sad and upset. She would feel all her effort to come to take the test was in vain. So this is the problem that I face.”* (Private-non-profit laboratory technician, rural primary health centre).

However, the women, who arrived in time for the test with the government employed laboratory technician, could still be turned down if they were not in the fourth, sixth or eight month of pregnancy. A 22 year old woman, five months pregnant with her second child, said she had been tested when pregnant with her first child. But in this pregnancy she had received contradicting advice from the HCPs about when to do the test: *“Last week a neighbour of mine did the test. They [the HCPs] told her that you have to do the sugar test in the fifth month… and [they] told me to go for the test”*. Arriving at the health centre, she was refused the test by the laboratory technician, who blamed her she had missed testing in the fourth month of pregnancy: *“He told me that only the sixth month women should do the test and not the fifth month women. It made me sad. If I don’t take the test this month, I will have to come back the next month.”* (Woman no. 21, rural area). The laboratory technician confirmed in an interview that he would assign new testing dates for all women coming for testing at incorrect gestational age. Thus, strict interpretations of guidelines and unaligned advice given by HCPs sometimes posed barriers for the pregnant women to initiate the test.

#### 2.b). Completing the test: Drinking glucose solution, drawing of blood and waiting

Some women, who initiated the test, failed to complete it. For instance, if they vomited after drinking the glucose solution some women were told to go home and return for a new test another day. Furthermore, in practice, the test would take a whole day to complete with travelling, drinking the glucose solution, drawing of blood, waiting at least two hours for the test to be completed, and travelling back. Urban as well as rural women found the waiting time very long and arduous, and some rural women complained about the facilities at the health centre being crowded, dirty, and with too few and sometimes rude staff: *“They are doing it for our good only, but at times I think if I have to go in this hot sun for all these tests. I feel it hard. I would feel giddy. I would feel as of vomiting. I would start at 7 and there would be a big crowd of 50 women, who would be waiting to do the tests.”* (Woman no. 18, rural area). For rural women, the test being free of charge was the main reason for attending the specific centre. In contrast, the urban women had few complaints about the physicality of the centre, but still found the waiting time long: *“I didn’t know I would be dedicating a whole day for this, but it looks like I have.”* (Woman no. 4, urban area).

#### 2.c). Getting the test report and potential diagnosis in a timely manner: Awareness, waiting and returning

After completing the test, there was still some ‘doing’ left. At the rural government centre, the laboratory technicians would inform the pregnant women of the test results via phone, through their village health nurse, or more commonly tell the women to collect the test report at the next ANC visit, typically between a week to a month later. Being informed about the results meant being told if the test results ‘were normal or not’. There was no mentioning of this in the government order, and one of the laboratory technicians described the procedure he followed:

*“If the patients wait on the same day for the results it would become late… If it’s an abnormal case [GDM] we would first inform the village health nurse to tell the woman to come, and we would then refer the woman to the General Hospital [in Chennai] on the same day... The other pregnant women would collect it when they come for the next check-up.”* (Government laboratory technician, rural centre).

However, in a few cases it took longer before the women were informed. Among the reasons for this were that the women had not been informed about how and when to collect the report; wherefore they were not aware that they were supposed to return. Some women had been told to return to the health centre, but found it difficult to repeat all the required arrangements.

At the private centre the women would get a 3–5 min consultation with the doctor, who would inform the woman whether the results were high or not, give instructions for what to do next and answer any questions the woman or her accompanying family member would have. However, this required that the woman would stay at the health centre after the test and wait typically another 1.5–2 h for the blood samples to be analysed and for consultation with the doctor. It was observed that some women left the health centre after completing the test, without waiting for the report. Informal conversations during those observations indicated that they did so because they had to return to work or more often out of considerations for the accompanying person.

## Discussion

### The complex screening process

This study explored the factors that influence the GDM screening process in TN, India. Limited research has been conducted on the factors influencing GDM screening initiation and completion in low and middle resource settings [9], and to our knowledge this is the first study, which includes pregnant women’s own perspectives.

Similarly to *Mol’s* work, we have shown that the GDM screening process requires a substantial amount of ‘doing’ and navigation of the health system by pregnant women, who have to go through several phases and sub-steps, each of which is influenced by several factors within and outside the health system. Other studies of maternal health have proposed similar steps-based frameworks for understanding barriers to health services use. Thus, ‘the three phases of delay’ framework suggested by *Thaddeus and Maine,* to understand barriers for seeking ‘institutionalised birth’, [33] describes three delay-steps: (i) dealing with the decision-making process involved in seeking health care, (ii) reaching a health facility and (iii) receiving adequate care once at the facility. Our study indicates that completion of GDM screening requires attendance of diverse services, at specific places at specific time points, including several repetitions, making it much more complex to successfully complete the screening procedure than the chronological ‘delay model’ suggest. These findings stress the importance of minimising and aligning the required ‘doing’ in screening and maternal health services. Complex and unaligned systems will surely fail to achieve the objective that all women should be tested for hyperglycaemia during pregnancy as stated by the International Federation of Obstetrics and Gynecology in their document on GDM [34].

The GDM diagnostic criteria currently being endorsed in TN by the Government do not require the women to be fasting and only requires one blood sample [16, 35]. It is, thus, a simpler process than many other existing criteria and screening approaches. Concerns about the feasibility of the main international guidelines in low and middle income countries have been raised by a number of groups [36–38], and this study further stresses the importance of guidelines and diagnostic criteria which are simple and feasible on the ground.

### Women’s perception of the screening process

In an earlier study from our group, challenges for GDM screening were investigated from the point of view of implementing partners of GDM projects in low and middle income countries [37]. Among the challenges identified were difficulties in screening women during the recommended time period, challenges in testing women in the fasting state, scarcity of test consumables, lack of equipment, and the screening procedure being too time consuming [37]. From our current study, it is clear that timing and waiting issues are considered problematic by both the pregnant women and HCPs. If the screening process cannot be shortened, it could be better utilised, e.g. by providing useful prenatal care. Also, the study showed the immense importance of HCPs’ abilities to properly inform and communicate with women: discussion and dialogue between HCPs and women are very important facilitators for screening.

But equally important is the ability of women to activate and draw on social networks in order to complete the screening process. Social network and support as facilitators for health seeking have also been identified in studies focusing on other diseases and conditions [39–43]. The importance of this is further emphasised through our findings that ‘fear’ of not living up to social norms of being a good mother, with attached social stigma, was a central underlying motivating factor to attend the screening test. This can be corroborated by other studies from India, showing that women’s inability to give birth to a healthy baby can be associated with stigmatisation [44–46]. Thus, a simple focus only on the behaviour, attitude etc. of the individual seems insufficient, and efforts should be made to further engage families and the local communities in ensuring the health of pregnant women and in combatting social stigma.

In this study, we showed how women across urban and rural divides in TN are highly motivated and make great efforts to complete the GDM screening test. But the findings also suggest that women with weak social networks, little family support and with poor access to the health centre are particularly vulnerable for not completing the process. This is much in line with evidence on factors influencing the use of general maternal health care in India (19;24–26). To ensure that these women are tested for GDM and receive treatment if needed, it is important that the identified barriers are addressed and facilitators are strengthened. This also requires actions within the health system, with the rural health system in particularly need of strengthening. It could among other things be considered to further consolidate the role of village health nurses, e.g. by equipping them with a glucometer to perform screening at the village level – literally at the women’s doorstep, accompanying the pregnant women to the health centres, and further train them to communicate effectively with women, including delivering test results.

### Study limitations

Our aim with this study was to explore how the process of GDM screening in TN is navigated and experienced, and what may cause or prevent delays or completion throughout the GDM screening and diagnosis process. We therefore sought to ensure maximum variation of women’s living places, life circumstances, gestational age at screening, and experiences with the GDM screening process. All women interviewed had attended a health centre at least once for GDM screening and had, therefore, all been able to overcome the initial barriers for screening. We did not recruit any women who had not attempted to attend the test at all. These women are likely facing even more pronounced barriers than the ones reported here.

The study presents findings from an urban private diabetes centre and a rural government health centre. Time and resources did not allow for more diversity, such as urban government and rural private health centres; hence, we chose the two health care settings likely to illustrate the biggest contrasts for health seeking behaviour and systems resources.

Most interviews were conducted with the help of an interpreter, which inevitably introduces some challenges, though the translator can also act as a co-researcher, adding local knowledge and assisting the interpretation of data [47]. This was enhanced by careful training of the interpreter, discussions after each interview and of the verbatim transcriptions and translation of the Tamil language into English.

## Conclusions

Our study investigated factors that influence the timely initiation and completion of the GDM screening process in an urban and a rural setting in TN. Factors both within and outside the health system were identified, including getting right information from HCPs, clinic timings, characteristics of the test, availability of transport, social network and support, and social norms and cultural practices. These factors should receive more attention in the future efforts to continue improving and expanding the GDM services in TN and the rest of India. Minimising and aligning complex stepwise processes of prenatal care and GDM screening delivery; mitigating barriers of waiting, timing and communication; and promoting social supportive facilitators for the screening process are important for further improving and expanding GDM screening – not only in TN but in other similar low and middle income settings as well.

## Additional files

Additional file 1: Interview guide – pregnant women. Contains the semi-structured interview guide used for the interviews with pregnant women regarding screening and testing for GDM. (DOCX 21 kb)

Additional file 2: Interview guide – HCP. Contains the semi-structured interview guide used for the interviews with health care providers regarding screening and testing for GDM. (DOCX 18 kb)

## Abbreviations

ANC: Antenatal care
DIPSI: Diabetes in Pregnancy Study Group India
GDM: Gestational diabetes mellitus
HCP: Health care provider
OGTT: Oral glucose tolerance test
TN: Tamil Nadu
