# Supplementary material for: Factors influencing timely initiation and completion of gestational diabetes mellitus screening and diagnosis - a qualitative study from Tamil Nadu, India
Source: BMC Pregnancy Childbirth. 2017 Aug 1;17:255. doi: 10.1186/s12884-017-1429-y (PMC5539632; doi:10.1186/s12884-017-1429-y)
Supplement: Supplementary file 1 — Interview guide – pregnant women. Contains the semi-structured interview guide used for the interviews with pregnant women regarding screening and testing for GDM. (DOCX 21 kb) [file 12884_2017_1429_MOESM1_ESM.docx]

## interview guide: with pregnant women screened and not screened for GDM

| **Theme** | **Main question** | **Probes** |
| --- | --- | --- |
| Personal characteristics | Could you tell me a bit about yourself? | Children? Work? Husband’s work? Education? Living with parents in laws? Where do you live? |
| Normal day/ daily life | Could you describe a normal day for you? | When do you get up?  What do you do during the day? Work? Shopping?  Taking care of children? Cooking? Do anyone help you with these things? Go to bed? |
| Before and going to the health centre | Could you describe to me what you did this morning? | When do you get up?  What did you do before going here?  How did you get here?  Taking care of children? Work? Cooking? |
| Healthy pregnancy | What is a healthy pregnancy in your opinion?  What is the biggest concern for a pregnant woman? | What can be done to ensure a healthy pregnancy?  In your opinion what kind of care do women need during pregnancy (from family)? What kind of health care should a pregnant woman have? Where do you get such care?  Why this type of care?  What happens if a pregnancy is not healthy?  Does going to the health center affect the pregnant woman’s mental wellbeing? |
| (ONLY ASK IF NOT IN A HEALHT CENTER)  Selection of health care services | What is important to you when selecting health care services? | Location? Staff? Waiting time? Cleanliness? |
| First ANC vist | Could you describe to me what happened at your first antenatal care (ANC) visit? | When did you leave your home? When did you arrive to the health centre?  What kind of examinations and test did they perform? How? When did you leave the health centre?  When in your pregnancy did you go for antenatal care the first time?  What made you go on that day? |
| Experience of being screened | Could you describe to me how you experienced the GDM screening test? | Could you describe what happened?  Could you describe your thoughts and emotions before and during the test?  Here you should probe a lot!  Angst, concerns, afraid, no idea, full trust in or just do what health staff says, having discussions with familiy etc etc. |
| Meaning of GDM | In your understanding, what does it mean to have GDM/diabetes during pregnancy? | Cause? Consequences? Treatment? for fetus, and mother and child later,  Will the pregnant woman continue to have diabetes after delivery?  Cost?  Has anyone told you anything about it? What did they say? |
| Knowledge and expectations of GDM test | Before coming here today what had you been told about the GDM test? | What had you been told to do? What had you been told would happen?  Who had told you? What did you think of this?  Did it live up to your expectations?  Did you have any hesitations or concerns about the screening test? If so, what were they?  What were the reasons you decided to come today and have the test/not have the test? |
| Social support | What do your family think about you going to antenatal care and having all these tests? | Do they all agree?  Have they said anything about it? What?  Is it something you discuss?  Who in your household would typically decide when and if you should go?  Is It important that a pregnant woman is accompanied when she goes to the health center? Why? |
| Risk perception | Did/do you think you might have diabetes?  What do you think will happen if you have diabetes? What would your family say? | Why/why not?  Probe: Affects on baby, affects on women, affects on pregnancy, affects on child health later etc. |
| Ideas for change | Do you think there is anything that could be done to make it more comfortable for a pregnant woman to come here and be tested for diabetes? |  |
| Intend to do it again | If you become pregnant again, would you come for the GDM screening again? | Why/why not? |
| Final question | Is there anything you would like to add regarding GDM screening that we have not talked about? |  |
