# Supplementary material for: Factors influencing timely initiation and completion of gestational diabetes mellitus screening and diagnosis - a qualitative study from Tamil Nadu, India
Source: BMC Pregnancy Childbirth. 2017 Aug 1;17:255. doi: 10.1186/s12884-017-1429-y (PMC5539632; doi:10.1186/s12884-017-1429-y)
Supplement: Supplementary file 2 — Interview guide – HCP. Contains the semi-structured interview guide used for the interviews with health care providers regarding screening and testing for GDM. (DOCX 18 kb) [file 12884_2017_1429_MOESM2_ESM.docx]

## Interview guide: with health care providers regarding GDM screening

| **Theme** | **Main question** | **Probes** |
| --- | --- | --- |
| Antenatal care organization | Could you describe to me how you have organized your antenatal care (ANC) services here at this health centre?  What services do you offer for pregnant women? | How big is the catchment area? i.e. how far are the women travelling to get here?  How many women attend antenatal care (ANC) on an average day?  What are the opening hours?  Can women come for antenatal care (ANC) every day or only some days? |
| Antenatal services | Could you describe to me briefly what steps do the pregnant woman attending an antenatal care (ANC) visit has to go through? | What tests are performed? (probes: urine test, blood pressure etc)  What types of health care providers will she see? |
| HCP own role | Could you describe what your tasks in antenatal care are? |  |
| Perceptions of barriers | What do you think are the reasons that some women do not attend antenatal care (ANC) or do not complete the recommended visits and tests? |  |
| Perceptions of facilitators | Is there anything that, in your experience, makes it more likely that a woman attends antenatal care (ANC) and completes the recommended visits and tests? |  |
| Organization of Gestational diabetes mellitus (GDM) screening | Could you describe to me how you have organized the GDM screening here at the clinic? | Who tells the women about the test?  Who performs the test?  Who tells the women the result of the test? |
| Introduction of GDM screening | When was GDM screening using blood test first introduced at your clinic? | How was it started? Why was it started?  What did you first think when you heard that GDM screening was to be introduced?  What changes did you have to make in the organization of services at the center?  How was introduction of GDM screening received by your clinic staff and colleagues? |
| Informing the women about GDM screening | How do you usually introduce the GDM screening test to the pregnant women? | What do you say or show?  Do you give information to her family as well or the community? |
| Reaction of the women | In your experience, how do the women usually receive the offer to be screened for GDM? | How do they react?  What do they say?  Do they ask questions? If so, what are they? |
| Refusal of test | Do you ever experience women refusing the test or are somehow reluctant to take it? | If so, why do you think that is?  Do you try to convince them to undertake the test? How do you do that? Why do you do that? |
| Completion of test | Do you ever have problems with initiating or completing the screening test? | If yes, what type of problems?  Why is that?  (e.g. not fasting, women vomiting, lack of consumables, woman not staying long enough for the test to be completed, no money for transport, not bothering about test/coming back). |
| Instructions in case of test failure | If the test can’t be completed, what instructions do you give the woman? | e.g. to come back another day? |
| Final question | Thank you very much for your comments. Do you have any questions about our research or final comments to the interview? | e.g. such as when and if the interviewers may come back and if feed-back will be given |
